# Down-regulation of miR-203 induced by *Helicobacter pylori* infection promotes the proliferation and invasion of gastric cancer by targeting CASK

**DOI:** 10.18632/oncotarget.2600

**Published:** 2014-10-18

**Authors:** Xiaoying Zhou, Guifang Xu, Chengqiang Yin, Wujuan Jin, Guoxin Zhang

**Affiliations:** ^1^ Department of Gastroenterology, First Affiliated Hospital of Nanjing Medical University, Nanjing, China; ^2^ First Clinical Medical College of Nanjing Medical University, Nanjing, China; ^3^ Department of Gastroenterology, Affiliated Drum Tower Hospital of Nanjing University Medical School, Nanjing, China

**Keywords:** miR-203, CASK, Helicobacter pylori, gastric cancer

## Abstract

Several microRNAs (miRNA) have been implicated in *H. pylori* related gastric cancer (GC). However, the molecular mechanism of miRNAs in GC has not been fully understood. In this study, we reported that miR-203 is significantly down-regulated in *H. pylori* positive tissues and cells and in tumor tissues with important functional consequences. Ectopic expression of miR-203 dramatically suppressed cell proliferation and invasion. We found that miR-203 strongly reduced the expression of CASK oncogene in GC cells. Similar to the restoring miR-203 expression, CASK down-regulation inhibited cell growth and invasion, whereas CASK over-expression rescued the suppressive effect of miR-203. These results can also be found in nude mice. In clinical specimens, CASK was over-expressed in tumors and *H. pylori* positive tissues and its mRNA levels were inversely correlated with miR-203 expression. Taken together, our results indicated that miR-203 functions as a growth-suppressive miRNA in *H. pylori* related GC, and that its suppressive effects are mediated mainly by repressing CASK expression.

## INTRODUCTION

Gastric cancer is the fourth most common cancer and second leading cause of cancer-related death worldwide [1]. To date, extensive studies of molecular mechanisms on GC have been investigated, however, few improvements on the early diagnosis of cancer have been made [2]. GC is supposed to developed according to a multistep process of carcinogenesis, which is strongly associated with the infection by the bacterial pathogen, *Helicobacter pylori* (*H. pylori*) [3]. *H. pylori*, which colonizes the stomach of more than 50% of the world population, has been classified as a class I carcinogen because of its causative role in the development of GC [4].

It has been reported that the development and progression of GC may be attributed to aberrant microRNAs (miRNAs) expression [5-8]. MiRNAs are small, non-coding RNAs that regulate the expression of target genes through translational repression or messenger RNA (mRNA) degradation [9]. For instance, a group of miRNAs, such as miR-10b [10] and miR-21 [11] are able to initiate invasion and metastasis in breast cancer, whereas some of other miRNAs including miR-200 family [12] and miR-126 [13] exert their inhibitory effect on invasion and metastasis in breast cancer. However, little is known about miRNA-203 functions in H. pylori induced GC.

Calcium/calmodulin-dependent serine protein kinase (CASK) is a member of membrane-associated guanylate kinase (MAGUK) family, a group of conserved cytoskeletal proteins that are composed of arrayed modular domains [14]. It is over-expressed in various cancers and it has been related to cell migration and invasion in studies [15, 16]. However, the role of CASK in GC has not been fully identified yet, especially in H. pylori induced GC.

In the present study, we demonstrated that miR-203 is significantly down-regulated in H. pylori positive tissues. Ectopic expression of miR-203 in GC cells suppressed invasion and migration *in vitro*. Furthermore, we identified and validated CASK gene as a novel and direct target of miR-203, as assessed by mutagenesis analysis of 3′-UTR of CASK gene and luciferase activity and showed that they play distinct roles in regulating H. pylori related GC development.

## RESULTS

### MiR-203 is aberrantly down-regulated in H. pylori positive tissues and cells

**Figure 1:**
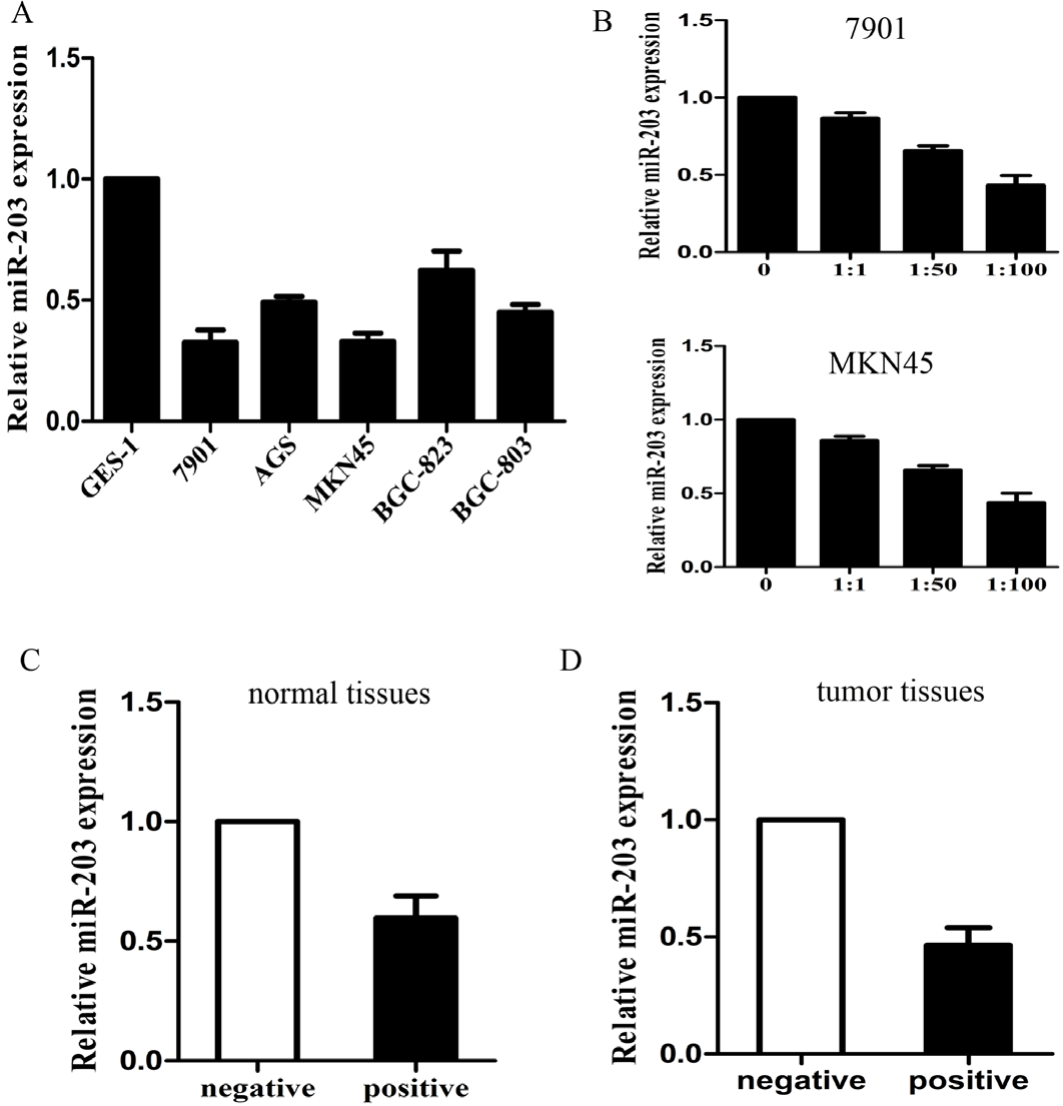
specimens and cells (A) miR-203 expression in 5 GC cell lines and immortalized GES-1 cells; (B) miR-203 expression in 7901 and MKN45 cells infected with different MOIs of H. pylori; (C) miR-203 expression in H. pylori positive and negative tumor tissues; (D) miR-203 expression in H. pylori positive and negative control tissues. (*p<0.05).

A panel of human GC cell lines was first analyzed to quantitate the expression level of miR-203. The results showed that the expression level of miR-203 was decreased in all 5 GC cell lines examined, compared with the immortalized non-tumorigenic cell line GES-1 (Figure 1A, p<0.05). 7901 and MKN45 cells displayed lowest miR-203. Consequently, we chose 7901 and MKN45 cells for further functional studies. We then infected 7901 and MKN45 cells with different MOIs of H. pylori (0, 1:1, 1:50, 1:100) and we found that miR-203 expression gradually decreased with increased MOIs (Figure 1B, p<0.05).

We further examined the expression level of miR-203 in 50 pairs of H. pylori positive and negative control specimens and in 50 pairs of H. pylori positive and negative tumor tissues. Consistent with the data obtained from GC cell lines, the average expression level of miR-203 by qRT-PCR was significantly lower in H. pylori positive tumor and normal tissues (Figure 1C and 1D). These results together showed that miR-203 was down-regulated in H. pylori infected state and its down-regulated was significantly associated with GC progression.

### Ectopic miR-203 inhibits growth and invasion of GC cells *in vitro*

**Figure2:**
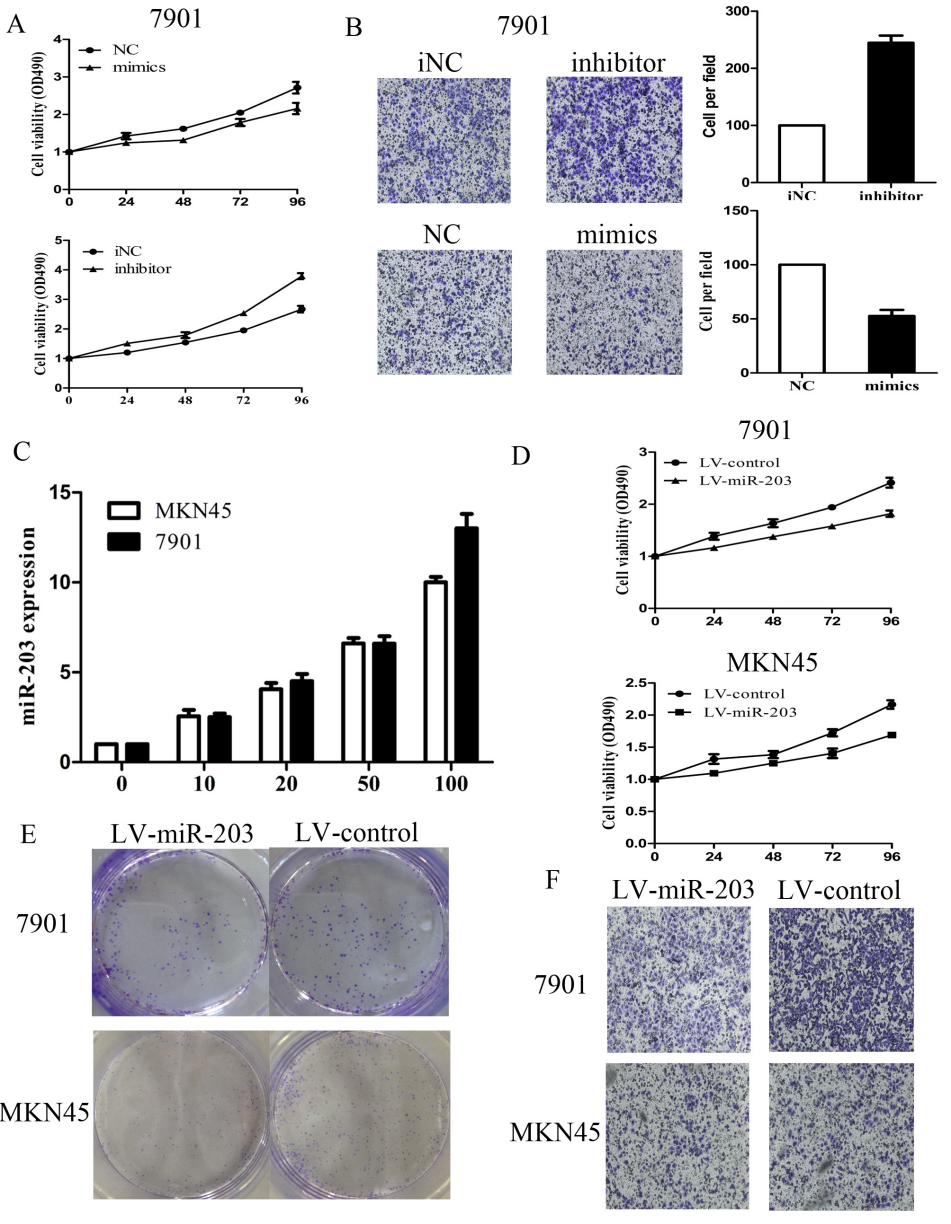
Effect of miR-203 on 7901 cell growth and invasion (A) effect of miR-203 on cell proliferation was measured by MTT assay after transfecting with miR-203 mimics/inhibitor; (B) effect of miR-203 on cell invasion was measured by transwell assay after transfecting with miR-203 mimics/inhibitor; (C) expression levels of miR-203 after 7901 and MKN45 cells were infected with LV-miR-203 at 5 different MOIs. (D) effect of miR-203 on cell proliferation was measured by MTT assay after transfecting with LV-miR-203 at MOI (100:1); (E) effect of miR-203 on cell proliferation was measured by clone assay after transfecting with LV-miR-203 at MOI (100:1); (F) effect of miR-203 on cell invasion was measured by transwell assay after transfecting with LV-miR-203 at MOI (100:1). (*p<0.05).

To explore the effect of miR-203 on cell growth and invasion, MKN-45 and 7901 cells were transiently transfected with miR-203 mimic or inhibitor, respectively. The expression of miR-203 validated after mimic or inhibitor transfection was shown in supplementary Figure 1A. As demonstrated in Figure 2A and supplementary Figure 1B, the results of MTT assay displayed that miR-203 significantly inhibited cell growth in 7901 cells and in MKN-45 cells (P<0.05), whereas miR-203 inhibitor promoted cell growth in these two cells (P<0.05). By contrast, negative control (NC) or inhibitor NC had no effect on cell growth, indicating that the effect caused by miR-203 was specific. Following observation of miR-203-mediated growth inhibition, we transfected 7901 or MKN45 cells with miR-203 mimic or inhibitor and examined cell invasion. Compared with inhibitor NC, cells transfected with miR-203 inhibitor displayed a significantly higher invasion rate, while cells transfected with miR-203 mimics inhibited the invasion compared with NC (Figure 2B and supplementary Figure 1C).

We next used lentiviral vectors to stably restore the expression of miR-203 in MKN-45 and 7901 cells and examined cell growth rate and invasion. We showed that the expression levels of miR-203 were increased in 7901 and MKN45 cells respectively in a dose-dependent manner and reached a very high level at MOI 100 (Figure 2C). Therefore, the same condition (MOI=100) was applied for further experiments. The growth inhibition induced by LV-miR-203 infection was similar to that induced by miR-203 mimic transfection (Figure 2D). As demonstrated in colony formation assay (Figure 2E), LV-miR203-infected 7901 and MKN45 cells displayed much fewer and smaller colonies compared with LV-con-infected cells. We also examined the cell invasion after transfection with LV-miR-203 and consistent with the previous results which showed that LV-miR-203 transfection significantly inhibit cell invasion (Figure 2F).

### CASK was a direct target of miR-203 in GC cells

**Figure3:**
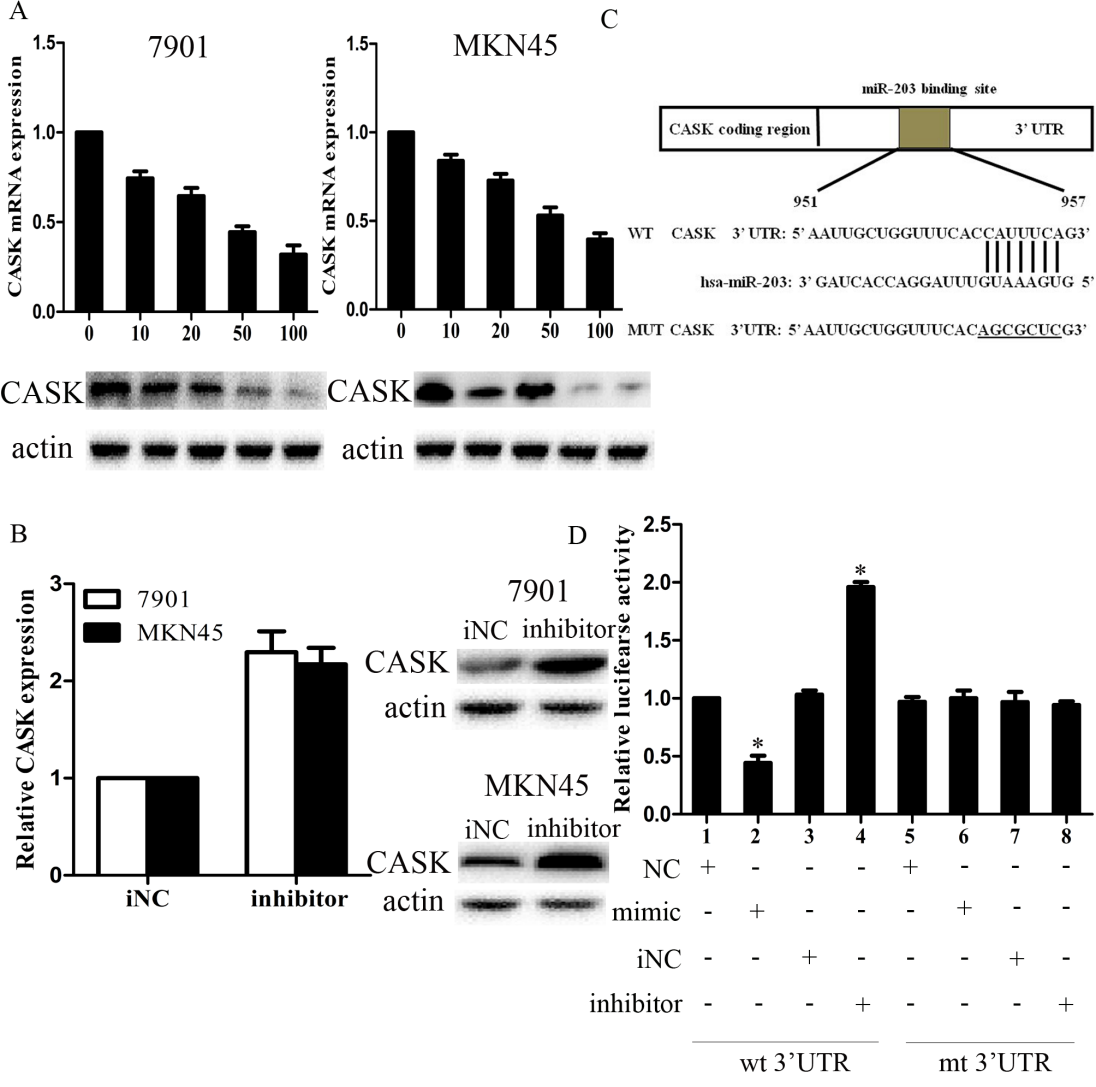
CASK was a direct target of miR-203 in GC cells (A) mRNA (upper) and protein (lower) levels of CASK after LV-miR-203 infection at different MOIs in 7901 and MKN45 cells; (B) mRNA (left) and protein (right) levels of CASK after 7901 cells were transfected with miR-203 inhibitor after 48 hours; (C) diagram of CASK 3′UTR-containing reporter constructs; (D) luciferase reporter assays in 7901 cells, with cotransfection of wt or mt 3′UTR and miRNA as indicated. (*p<0.05 compared with control).

To explore the mechanism of growth and invasion inhibition induced by miR-203, we investigated whether miR-203 could regulate CASK expression in GC cells. CASK had been reported to regulate GC progression; however, the status and function of CASK have never been studied in GC. We transfected 7901 and MKN45 cells with LV-miR203 at 5 different MOIs of 0, 10, 20, 50, and 100 and then examined CASK expression levels. As shown in Figure 3A, ectopic expression of miR-203 led to a dose-dependent decrease in CASK mRNA and protein levels. At MOI 100, both the mRNA and protein levels of CASK were decreased by approximately 70% to 80%. Moreover, inhibition of endogenous miR-203 by miR-203 inhibitor resulted in up-regulated expression of CASK in 7901 cells (Figure 3B).

We further performed luciferase reporter assay to determine whether miR-203 could directly target the 3′UTR of CASK in GC cells. The target sequence of CASK 3′UTR (wt 3′UTR) or the mutant sequence (mut 3′UTR) was cloned into a luciferase reporter vector (Figure 3C). 7901 cells were then transfected with wt or mut 3′UTR vector and miR-203 mimic. The results showed a significant decrease of luciferase activity when compared with miRNA control (Figure 3D). The activity of mut 3′UTR vector was unaffected by a simultaneous transfection with miR-203. Moreover, co-transfection with miR-203 inhibitor and wt 3′ UTR vector in 7901 cells led to a 2-fold increase of luciferase activity. Taken together, all these results strongly suggested that CASK was a direct target of miR-203 in GC cells.

**Figure4:**
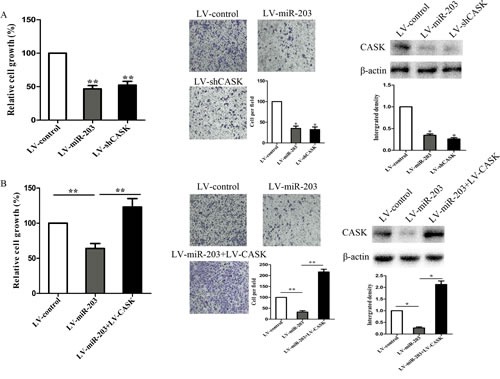
CASK was involved in miR-203-induced growth inhibition in 7901 cells 7901 cells were infected with LV-shCASK or LV-miR-203. (A) Cell growth rate was measured by MTT assay. (B) Cell invasion was measured by transwell assay. (C) Protein expression of CASK was measured by western blot analysis. 7901 were infected with LV-miR-203 for 72 hours, followed by infection with LV-CASK. (D) Cell growth rate was measured by MTT assay. (E) Cell invasion was measured by transwell assay. (E) Protein expression of CASK was measured by western blot analysis. (*p<0.05 compared with control).

To elucidate whether the growth and invasion suppressive effect of miR-203 was mediated by repression of CASK in GC cells, we performed gain-of-function and loss-of-function studies. Firstly, we silenced CASK to investigate whether the reduced expression of CASK could mimic the suppressive effect of miR-203. 7901 cells were infected with sh-CASK or LV-miR-203 and then we examined cell growth rate and invasion ability. As shown in Figure 4A, CASK knockdown led to significant cell growth and invasion inhibition, similar to those induced by miR-203 (P<0.01). Subsequently, we evaluated whether ectopic expression of CASK could rescue the suppressive effect of miR-203. 7901 cells were infected with LV-miR-203 for 72 hours and followed by infection with pcDNA-CASK, which encoded the full-length coding sequence without the 3′ UTR region. We showed that ectopic expression of CASK significantly rescued miR-203 induced cell growth and invasion inhibition (Figure 4B).

### MiR-203 suppressed tumor growth of GC cells in nude mice

**Figure5:**
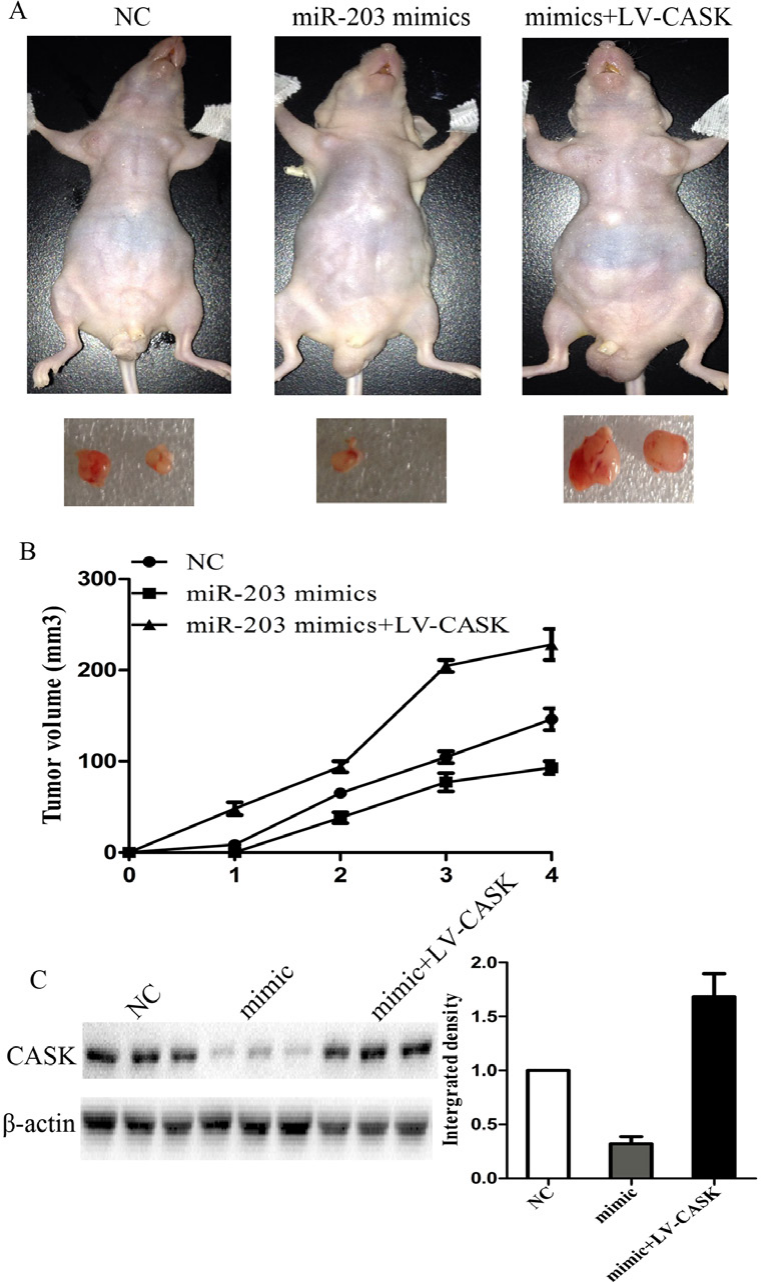
MiR-203 suppressed tumor growth of GC cells in nude mice A, 7901 cells were infected with LV-miR-203 or LV-miR-203 and LV-CASK and injected subcutaneously into nude mice. After 4 weeks, LV-miR-203-infected cells (middle) produced smallest tumors, while cells transfected with LV-miR-203 and LV-CASK produced largest tumors. (B) growth curve of tumor volumes. (C) protein expression levels of CASK in tumors formed. (*p<0.05 compared with control).

7901 cells transfected with LV-miR-203 or LV-miR-203 and pcDNA-CASK were injected subcutaneously into the nude mice. All the mice developed tumors at the end of the experiment. As compared with control, the average tumor volume of the LV-miR203-treated group was markedly reduced by more than 65% and LV-miR-203 and pcDNA-CASK treated group exhibited significantly larger tumors (Figure 5A and 5B). Then, we extracted RNA and protein from the tumors and found that miR-203 expression was significantly higher in LV-miR-203 treated group. CASK expression was significantly higher in LV-miR-203 and pcDNA-CASK treated group and lower in LV-miR-203 treated group as compared to control group (Figure 5C).

### CASK was up-regulated in H. pylori positive cells and tissues and inversely correlated with miR-203 levels

**Figure6:**
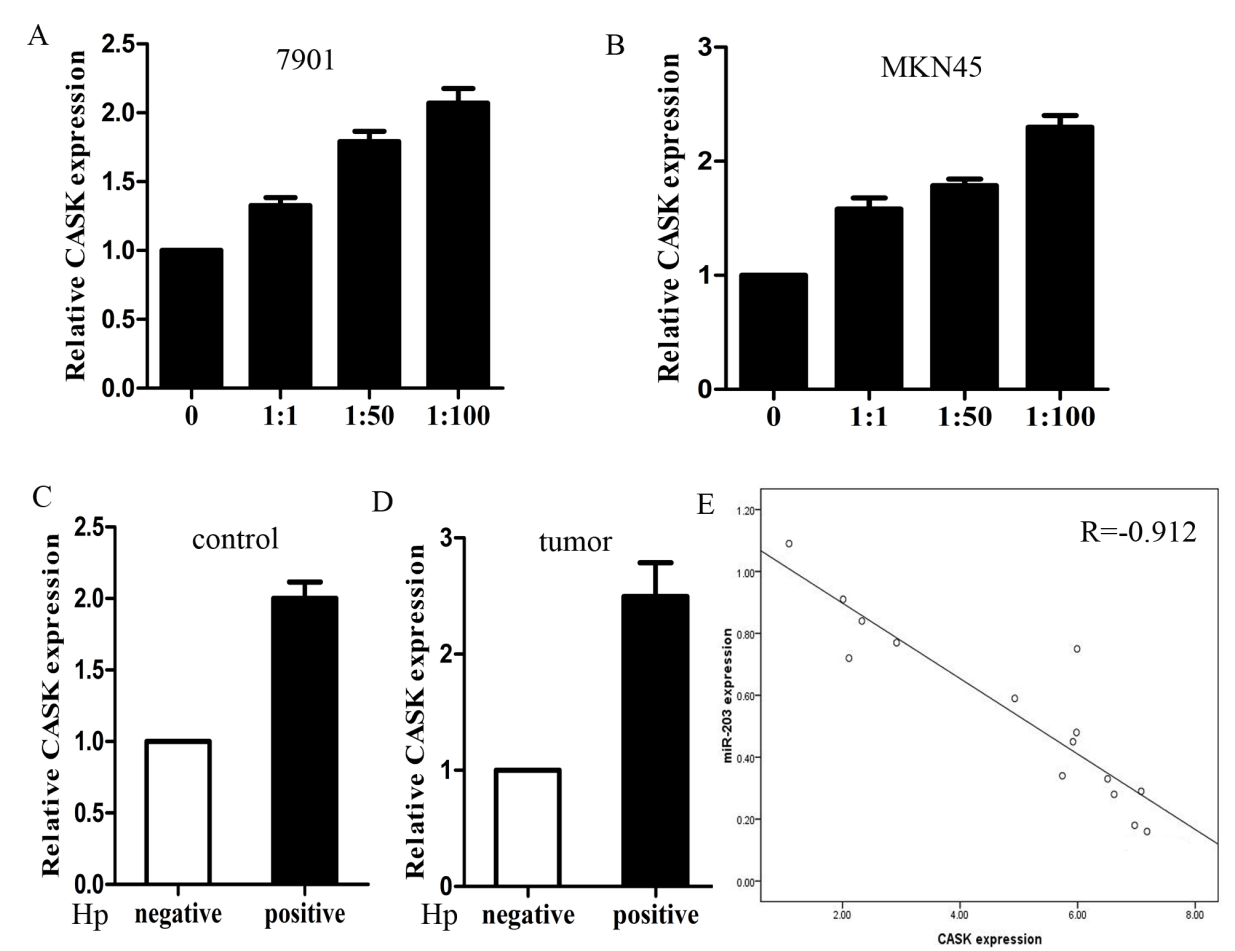
The expression of CASK was up-regulated in cell lines and clinical specimens (A) expression of CASK in different MOIs of H. pylori infected 7901 cells; (B) expression of CASK in different MOIs of H. pylori infected MKN45 cells; (C) expression of CASK in H. pylori positive and negative control tissues; (D) expression of CASK in H. pylori positive and negative tumor tissues; (E) a statistically significant inverse correlation between miR-203 and CASK levels in clinical specimens (Spearman's correlation analysis, r=-0.912, p=0.000) (*p<0.05 compared with control).

We further measured the mRNA levels of CASK in H. pylori positive and negative cells and specimens. The results showed that the average mRNA level of CASK was significantly higher in H. pylori infected cells and the expression increased with increasing MOIs (Figure 6A and 6B). We also found that CASK expressed significantly higher in H. pylori infected tumor and control tissues (Figure 6C and D). Then, we correlated CASK with miR-203 expression in the same GC specimens. As shown in Figure 6E, when CASK mRNA levels were plotted against miR-203 expression, a significant inverse correlation was observed (2-tailed Spearman's correlation, r=-0.912; P=0.000).

## DISCUSSION

H. *pylori* infection rate is increasingly higher nowadays, especially in developing countries [17]. Most clinical evidence suggested that H. *pylori* infection is related to GC, but the underlying molecular mechanism remained largely unknown [18]. Majority of cancer-related death is caused by metastasis and recent studies have demonstrated that miRNAs play an important role in the regulation of this process [19]. Previous studies have demonstrated that the expression profiling of miR-203 was tissue-specific and that it might have divergent functions depending on the tumor tissue or cell type. MiR-203 has been reported as a novel tumor and metastasis suppressor by directly targeting mRNA of PLD2 [20], Bmi-1 [21] and PKCα [22]. However, this was the first study to explore the underlying mechanism of H. pylori related gastric cancer. H. pylori infection might alter miRNAs expression through epigenetic regulations such as DNA methylation and histone modification. Further investigations are needed to figure out how H. pylori infection changed miR-203 expression. Here, we identified CASK as a new direct target of miR-203 and miR-203 exerts its tumor-suppressive function via down-regulating CASK oncogene.

In this study, we found that the expression of miR-203 in H. pylori positive specimens was significantly lower than that in negative tissues, regardless of normal or tumor tissues. We also infected cells with H. pylori of different MOIs and miR-203 was decreased accordingly. To further reveal the roles of miR-203 in GC cells, we tested the effect of miR-203 on cell growth and invasion. Our results showed that miR-203 could inhibit cell growth, colony formation and cell invasion and suppress tumorigenesis in a murine model of GC xenograft, suggesting its potential tumor suppressor role in H. pylori induced GC. The data were similar to the findings in glioblastoma and prostate cancer, in which miR-203 was down-regulated, and ectopic expression of miR-203 suppressed cell proliferation and induced a G1-phase arrest by targeting PLD2 [20]. However, miR-203 was amplified and could promote cell growth and drug resistance in colorectal cancer [23]. The controversial results suggested that the role of miR-203 was possibly tumor specific and highly dependent on its targets in different cancer cells. Indeed, the tissue- and time-dependent expression of miRNAs influenced protein translation during distinct cellular processes, and the aberrant expression of their target genes affected different biological pathways with diverse functions.

It is well known that an average miRNA has approximately 100 target sites and regulates a large fraction of protein coding genes, which form a regulatory network [24]. To further explore the molecular mechanisms of growth inhibition induced by miR-203, we searched the potential target of miR-203 through Targetscan and found that CASK could match the sequence of miR-203. We further identified the association between miR-203 and CASK through luciferase activity. The luciferase activity was significantly lower in cells transfecting CASK wt 3′UTR, suggesting that a direct interaction between miR-203 and its targeted gene. CASK and members of the MAGUK family have recently been recognized as important organizing proteins in the cortical protein networks [25]. Wang et al [26] found that CASK was significantly up-regulated in human esophageal carcinoma and was associated with the poor prognosis of cancer. Lucrecia et al [25] found that CASK interaction with Cx43 and their co-expression affects cell migration. However, there are few studies investigating the role of CASK in GC, especially in H. pylori-induced GC. In our study, we found that CASK mRNA expression was significantly higher in H. pylori infected cells than that in control cells. Furthermore, we silenced the CASK expression in GC cells and found that the cell functions were comparable when cells transfecting with miR-203 mimics or sh-CASK. We then overexpressed CASK in cells and found that CASK overexpression could reverse the effect of miR-203 mimics. These results together suggested that miR-203 could inhibit cell growth and invasion through CASK repression.

In addition to the oncogenic effects of CASK in GC cells, for the first time, we showed that was naturally up-regulated in H. pylori infected GC and normal specimens and inversely correlated with miR-203 levels, suggesting that CASK might play important roles in GC tumorigenesis. We suggested that the overexpression of CASK in GC held significant promise for the advancement of cancer therapy, either in terms of improving diagnosis or predicting prognosis. Although this claim awaited further validation on larger sizes of samples, CASK dysregulated levels might prove valuable as prognostic markers, especially as CASK overexpression seemed to be strongly associated with the poor prognosis in both metastatic breast and prostate cancers.

In conclusion, our evidence indicated that miR-203 was down-regulated, while CASK was up-regulated in H. pylori induced GC. Ectopic miR-203 expression decreased CASK expression at the mRNA and protein levels in the GC cell line. We also demonstrated that miR-203 was directly bound to the 3′-UTR of CASK. MiR-203 expression inhibited GC growth and invasion via repression of CASK. Furthermore, the miR-203-CASK pathway that we identified may be exploited in a therapeutic approach for the treatment of H. pylori infection induced GC.

## MATERIALS AND METHODS

### Clinical Samples

Gastritis or GC patients with or without H. pylori infection had undergone gastroscope at The First Affiliated Hospital of Nanjing Medical University, Jiangsu Province, China from March 2013 and March 2014 were enrolled. The GC samples were collected, immediately snap frozen in liquid nitrogen, and stored at −80 °C until RNA extraction. The samples were identified as H. pylori positive when rapid urease test was positive. These selected patients were also confirmed by 13C breath test. This study was approved by the Ethical Committee of the first affiliated hospital of Nanjing Medical University, and every patient had written informed consent.

### Cell culture

GC 7901 and MKN45 cells were obtained from ATCC (Shanghai, China) and were cultured in RPMI-1640 plus 10% heat-inactivated fetal bovine serum (FBS), 100 mg/mL streptomycin, and 200 U/mL penicillin (Life Technologies).

### Quantitative RT-PCR and Western blot analysis

Total RNA was extracted using Trizol (TAKARA, Japan). Levels of mature miR-203 were measured using TaqMan MicroRNA Assay (Applied Biosystems) by normalizing to the levels of U6. SYBR Green PCR Kit (TAKARA, Japan) was used to quantify the mRNA levels of CASK by normalizing to GAPDH. The primers of reverse transcription and PCR were listed in supplementary Table 1. The PCR reactions were performed and analyzed using ABI Step-one system. The relative expression ratio of miR-203 in paired tissues and cells was calculated by the 2^−ΔΔCT^ method. Western blots were performed as described previously. Briefly, total protein was separated on a precast 8% polyacrylamide gel and blotted with antibodies for CASK (diluted 1:1000, cell signaling technology) and β-action (diluted 1:1000, cell signaling technology). Densitometric analysis of protein bands was performed via Image J software.

### Dual-luciferase assays

For CASK, fragments containing the predicted binding sites for miR-203 at the 3′-untranslated regions (UTR) were amplified from 7901 genomic DNA by PCR. PCR products were cloned downstream of the firefly luciferase gene in pGL3 to obtain wild-type pGL3-CASK-3′UTR. To construct mutant vectors, putative miR-203 binding sites in CASK 3′-UTR were mutated (Genepharma, Shanghai). All inserts were sequenced to verify the mutations. Primers used for PCR and sequencing are presented in Supplementary Table S1. For luciferase assays, 7901 cells were plated in 24-well plates and, 24 hours later, co-transfected with 30 nmol/L miR-203 mimic or inhibitor or their respective negative controls, 1mg pGL3-CASK or vectors containing wild-type or mutant CASK 3′UTR, together with 0.5 mg pGL3-Renilla expressing vector (transfection control). Forty-eight hours later, luciferase activities were measured using Dual Luciferase Reporter Assay Kit (Promega) on a Gen-Probe chemiluminometer.

### MTT and invasion assays

For MTT assays, 5,000 cells were seeded in 96-well plates and transfected with various vectors for 72 hours using Lipofectamine 2000. Then, cells were stained with 100mL MTT dye (0.5 mg/mL) for 2 hours at 37°C, followed by adding 50μL dimethyl sulphoxide (DMSO). The optical density was measured at 590 nm with a microplate reader (Bio-Rad). For invasion assays, GC cells were transfected with miR-203 mimic or inhibitor or their respective controls for 48 hours, after which 50,000 cells in serum-free medium were seeded in the top chamber of 24-well transwell units (BD Pharmingen) with RPMI-1640 containing 15% FBS added to the bottom chambers. Cells were allowed to migrate for 20 hours at 37°C, and then cells in the top chambers were removed and cells that invaded into the bottom chambers were fixed, stained, and quantified.

### Clonal formation assay

For clonal experiments, transfected cells were seeded at low density (100 cells/well) in a 6-well plate and allowed to grow until visible colonies appeared. Clones were counted within 2 weeks.

### Oligonucleotides, plasmids, and transfection

Lipofectamine 2000 (Invitrogen) was used to transfect GC cells with 30 nmol/L miR-203 mimic or inhibitor or their respective non-targeting negative control oligonucleotides (Genepharma) according to the manufacturer's instructions. 3′-UTR sequence of CASK which was predicted to interact with miR-203 or a mutant sequence with the predicted target sites were inserted into the KpnI and SacI sites of pGL3 promoter vector (Invitrogen). They were named pGL3-CASK and pGL3-CASK-mut.

The DNA fragments corresponding to CASK or miR-203 were amplified from human genomic DNA and were listed in supplementary table 1. Then, they were cloned into pLVTHM lentiviral vector. Virus packaging was performed in HEK293T cells using lipofectamine 2000. The 7901 cells were transduced with pLV-SOCS2. Forty-eight hours after infection, 2 mg/ml of puromycin was added to the media for 2 weeks to select the cells infected with the lentivirus. The empty lentiviral vector LV-control was used as a control.

### Statistical analysis

All statistical analyses were performed using SPSS 17.0. All data were expressed as the mean ± SD of at least 3 independent experiments. The differences between groups were analyzed using the Student's t-test; P<0.05 was considered to indicate statistically significant differences.

## SUPPLEMENTARY TABLES AND FIGURE


