# Supplementary material for: Down-regulation of miR-203 induced by Helicobacter pylori infection promotes the proliferation and invasion of gastric cancer by targeting CASK
Source: Oncotarget. 2014 Oct 18;5(22):11631–40. doi: 10.18632/oncotarget.2600 (PMC4294334; doi:10.18632/oncotarget.2600)
Supplement: Supplementary file 1 [file oncotarget-05-11631-s001.pdf]

Down-regulation of miR-203 induced by Helicobacter pylori infection promotes the proliferation and invasion of gastric cancer by targeting CASK

Supplementary Material

Supplementary Table 1: Primers for qRT-PCR analysis

Name		Sequences
qPCR primers		
miR-203 Reverse		GTCGTATCCAGTGCAGGGTCCGAGGTATTCGCACTGGATACG ACCGCCAATA
miR-203	Sense	5’-GGGGTGAAATGTTTAGGAC-3’
	Anti-sense	5’-CAGTGCGTGTCGTGGAGT-3’
U6	Sense	5’- CTCGCTTCGGCAGCACA-3’
	Anti-sense	5’-AACGCTTCACGAATTTGCGT-3’
CASK	Sense	5'-TTGAAATCGTAAAGCGAGCTGA-3'
	Anti-sense	5'-CAGTAGCGTAGAGCTTCCAGTA-3'
GAPDH	Sense	5'- GGTGAAGGTCGGAGTCAACGGA-3'
	Anti-sense	5'- GTCATGGATGACCTTGGCCAGG-3'
pLV-miR-203		GTGTTGGGGACTCGCGCGCTGGGTCCAGTGGTTCTTAACAGTTCAA CAGTTCTGTAGCGCAATTGTGAAATGTTTAGGACCACTAGACCCGGC GGGCGCGGCGACAGCGA
pLV-CASK		ATGGCCGACGACGACGTGCTGTTCGAGGATGTGTACGAGCT GTGCGAGGTGATCGGAAAGGGTCCCTTCAGTGTTGTACGAC GATGTATCAACAGAGAAACTGGGCAACAATTTGCTGTAAAAA TTGTTGATGTAGCCAAGTTCACATCAAGTCCAGGGTTAAGTA CAGAAGATCTAAAGCGGGAAGCCAGTATCTGTCATATGCTGA AACATCCACACATTGTAGAGTTATTGGAGACATATAGCTCAGA TGGAATGCTTTACATGGTTTTCGAATTTATGGATGGAGCAGATC TGTGTTTTGAAATCGTAAAGCGAGCTGACGCTGGTTTTGTGTA CAGTGAAGCTGTAGCCAGCCATTATATGAGACAGATACTGGAA GCTCTACGCTACTGCCATGATAATAACATAATTCACAGGGATGT GAAGCCCCACTGTGTTCTCCTTGCCTCAAAAGAAAACTCGGC ACCTGTTAAACTTGGAGGCTTTGGGGTAGCTATTCAATTAGGGG AGTCTGGACTTGTAGCTGGAGGACGTGTTGGAACACCTCATTTT ATGGCACCAGAAGTGGTCAAAAGAGAGCCTTACGGAAAGCCTG TAGACGTCTGGGGGTGCGGTGTGATCCTTTTTATCCTGCTCAGTG GTTGTTTGCCTTTTTACGGAACCAAGGAAAGATTGTTTGAAGG CATTATTAAAGGAAAATATAAGATGAATCCAAGGCAGTGGAGC

CATATCTCTGAAAGTGCCAAAGACCTAGTACGTCGCATGCTGATG
CTGGATCCAGCTGAAAGGATCACTGTTTATGAAGCACTGAATCAC
CCATGGCTTAAGGAGCGGGATCGTTACGCCTACAAGATTCATCTT
CCAGAAACAGTAGAGCAGCTGAGGAAATTCAATGCAAGGAGGA
AACTAAAGGGTGCAGTACTAGCCGCTGTGTCAAGTCACAAATTC
AACTCATTCTATGGGGATCCCCCTGAAGAGTTACCAGATTTCTCC
GAAGACCCTACCTCCTCAGGACTTCTAGCAGCAGAAAGAGCAGT
CTCACAGGTGCTGGACAGCCTGGAAGAGATTCATGCGCTTACAGA
CTGCAGTGAAAAGGACCTAGATTTTCTACACAGTGTTTTCCAGGA
TCAGCATCTTCACACACTACTAGATCTGTATGACAAAATTAACA
CAAAGTCTTCACCACAAATCAGGAATCCTCCAAGCGATGCAGT
ACAGAGAGCCAAAGAGGTATTGGAAGAAATTTCATGTTACCCT
GAGAATAACGACGCAAAGGAACTAAAGCGTATTTTAACACAAC
CTCATTTCATGGCCTTACTTCAGACTCACGACGTAGTGGCACATG
AAGTTTACAGTGATGAAGCATTGAGGGTCACACCTCCTCCCACC
TCTCCCTATTTAAACGGCGATTCTCCAGAAAGTGCTAACGGAGA
CATGGATATGGAGAATGTGACCAGAGTTCGGCTGGTACAGTTTC
AAAAGAACACAGATGAACCAATGGGAATCACTTTAAAAATGAA
TGAACTAAATCATTGTATTGTTGCAAGAATTATGCATGGGGGCA
TGATTCACAGGCAAGGTACACTTCATGTTGGTGATGAAATTCGA
GAAATCAATGGCATCAGTGTGGCTAACCAAACAGTGGAACAAC
TGCAAAAAATGCTTAGGGAAATGCGGGGGAGTATTACCTTCAAG
ATTGTGCCAAGTTACCGCACTCAGTCTTCGTCCTGTGAGGACTTG
CCATCAACTACCCAACCAAAAGGACGACAGATCTATGTAAGAGCA
CAATTTGAATATGATCCAGCCAAGGATGACCTCATCCCCTGTAAA
GAAGCTGGCATTCGATTCAGAGTTGGTGACATCATCCAGATTATT
AGTAAGGATGATCATAATTGGTGGCAGGGTAAACTGGAAAACTCC
AAAAATGGAACTGCAGGTCTCATTCCTTCTCCTGAACTTCAGGAA
TGGCGAGTAGCTTGCATTGCCATGGAGAAGACCAAACAGGAGCA
GCAGGCCAGCTGTACTTGGTTTGGCAAGAAAAAGAAGCAGTACA
AAGATAAATATTTGGCAAAGCACAATGCAGATCTTGTCACATATGA
AGAAGTAGTAAAACTGCCAGCATTCAAGAGGAAAACACTAGTCT
TATTAGGCGCACATGGTGTTGGGAGAAGACACATAAAAAACACT
CTCATCACAAAGCACCCAGACCGGTTTGCGTACCCTATTCCACAT
ACAACCAGACCTCCAAAGAAAGACGAAGAAAATGGAAAGAATT
ATTACTTTGTATCTCATGACCAAATGATGCAAGACATCTCTAATAA
CGAGTACTTGGAGTACGGCAGCCACGAGGATGCGATGTATGGGA
CAAAACTGGAGACCATCCGGAAGATCCACGAGCAGGGGCTGATT
GCAATACTGGACGTGGAGCCTCAGGCACTGAAGGTCCTGAGAA
CTGCAGAGTTTGCTCCTTTTGTTGTTTTCATTGCTGCACCAACTAT
TACTCCAGGTTTAAATGAGGATGAATCTCTTCAGCGTCTGCAGAA
GGAGTCTGACATCTTACAGAGAACATATGCACACTACTTCGATCT
CACAATTATCAACAATGAAATTGATGAGACAATCAGACATCTGGA
GGAAGCTGTTGAGCTCGTGTGCACAGCCCCACAGTGGGTCCCTGT

CTCCTGGGTCTATTAG

Supplementary Table 2 Basic characteristics of the enrolled tumor patients

Basic characteristics	Hp positive	Hp negative	P value
Number	50	50	
Gender (male/female)	29/21	27/23	0.840
Age	51.02±2.3	49±3.8	0.682
TNM stage (1, 2/3, 4)	33/17	29/21	0.537
Metastasis (M0/M1)	39/11	36/14	0.645
Differentiation			
Well	30	28	0.840
Moderate/poor	20	22	

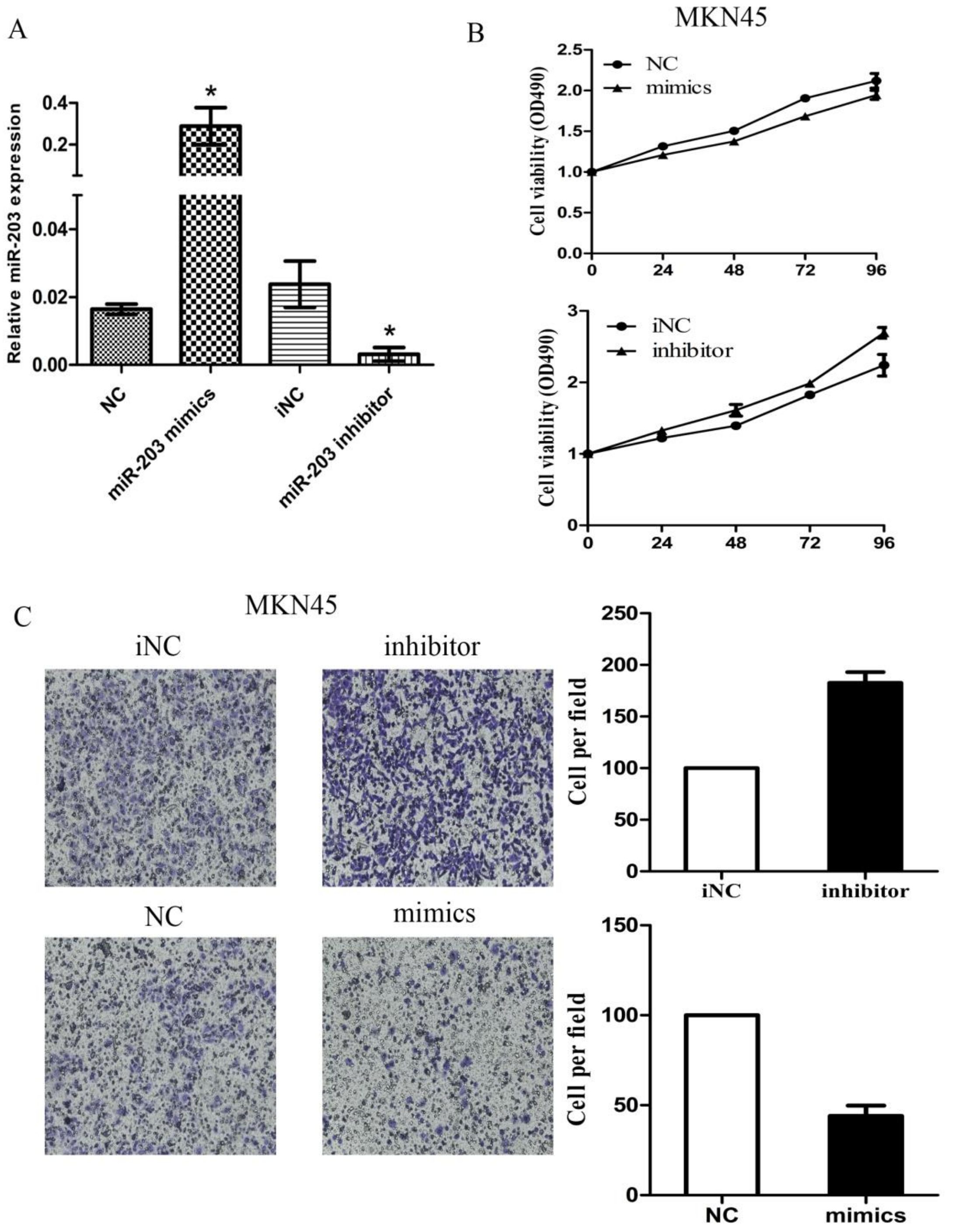

Supplementary Figure 1: (A) miRNA expression validated by qRT-PCR of miRNA mimic or inhibitor transfection; (B) effect of miR-203 on cell proliferation was measured by MTT assay after transfecting with miR-203 mimics or inhibitor in MKN45 cells; (C) effect of miR-203 on cell invasion was measured by transwell assay after transfecting with miR-203 mimics or inhibitor in MKN45 cells (*p<0.05 compared with control).
